# Correspondence about the article: Trends on the Brazilian asthma mortality rate: a call for a standardized protocol analysis from the DATASUS databases

**DOI:** 10.36416/1806-3756/e20240162

**Published:** 2024-11-26

**Authors:** Marcos Otávio Antunes, Frederico Friedrich, Paulo Pitrez

**Affiliations:** 1. Faculdade de Medicina, Pontifícia Universidade Católica do Rio Grande do Sul, Porto Alegre (RS), Brasil.; 2. Santa Casa de Porto Alegre, Pavilhão Pereira Filho, Porto Alegre (RS), Brasil.

## DEAR EDITOR:

We appreciate the recent article by Pinheiro et al. (2024), entitled “Asthma in the Brazilian Unified Health Care System: an epidemiological analysis from 2008 to 2021”, published in the *Jornal Brasileiro de Pneumologia*, which provides valuable insights into the epidemiology of asthma in Brazil.^1^ However, we have some concerns regarding the interpretation of the asthma mortality data, which warrants further attention.

Pinheiro et al.^1^ reported lower mortality rates than those previously documented in Brazil, which totaled 2,047 in 2013.^2^ Between 2008 and 2021, Pinheiro et al.^1^ reported 8,497 deaths from asthma, whereas other official mortality data sources indicate that this number was approximately 34,000. Upon reproducing this data, we found that the values presented by the authors were derived from TabNet, which focuses exclusively on deaths related to hospitalizations. Relying on data from this platform may lead to a significant underestimation of asthma-related deaths, as it does not account for deaths occurring outside the hospital setting, including those in non-hospital healthcare facilities. 

The presentation of this data using the term “mortality rate” implies that all deaths from the disease in Brazil are included, as already described in both national and international literature. For this reason, we consider the use of underlying cause of death data, available in the Mortality Information System (SIM), to be the most appropriate choice to avoid underreporting. Furthermore, the lack of population adjustment based on the most recent IBGE census (2022) may have led to an overestimation of the population, thereby impacting the mortality rate calculation, as the 2010 projections referenced in the study have an estimated error of approximately 10 million inhabitants. 

Our results, submitted for publication in this journal in April 2024 (ID: JBPNEU-2024-0138), indicate that after a long-term historical decline, there was an increasing trend in asthma mortality after 2014. We highlight that the data analyzed in our report (detailed information available at: https://github.com/mobrant94/asthma_mortality) took the following key factors into account: 1) Brazilian population growth from 2014 to 2021; 2) Exclusion of cases related to the COVID-19 pandemic in 2020 and 2021; and 3) Exclusion of the number of registered deaths in individuals aged ≤ 6 years. Our findings in terms of numbers and mortality rates align with the trends reported by the World Health Organization (WHO) Mortality Database platform for the period between 2014 and 2020.^3^ We recommend that future research on asthma mortality in Brazil utilize the same data sources for general asthma mortality, specifically the DATASUS Mortality Information System (SIM), which encompasses all causes of death and allows for a reliable analysis of mortality. 

In summary, it is important that future epidemiological studies on asthma mortality employ standardized methods and reliable data sources to avoid misinterpretations. We appreciate the opportunity to discuss these issues and hope to contribute to a better understanding of asthma mortality trends in Brazil.
