# Supplementary material for: Correspondence about the article: Trends on the Brazilian asthma mortality rate: a call for a standardized protocol analysis from the DATASUS databases
Source: J Bras Pneumol. 2024 Nov 26;50(6):e20240162. doi: 10.36416/1806-3756/e20240162 (PMC11601066; doi:10.36416/1806-3756/e20240162)
Supplement: Related texts

# Author’s Reply about the article: Asthma in the Brazilian Unified Health Care System: An Epidemiological Analysis from 2008 to 2021.

**Authors:** David Halen Araújo Pinheiro, João Victor Hermógenes de Souza, Alberto Fernando Oliveira Justo, Regina Maria Carvalho-Pinto, Fabiano Francisco de Lima, Celso R. F. Carvalho

**Affiliations:** 1. Departamento de Fisioterapia, Faculdade de Medicina, Universidade de São Paulo, São Paulo (SP), Brasil.; 2. Laboratório de Fisiopatologia do Envelhecimento, Departamento de Clínica Médica, Universidade de São Paulo, São Paulo (SP), Brasil.; 3. Divisão de Pneumologia, Instituto do Coração - InCor - Hospital das Clinicas, Faculdade de Medicina, Universidade de São Paulo, São Paulo (SP), Brasil.

We received with great interest the correspondence “Trends on the Brazilian asthma mortality rate: a call for a standardized protocol analysis from DATASUS databases” (ID: JBPNEU-2024-0162), which includes comments from Antunes et al. regarding our study, “Asthma in the Brazilian Unified Health Care System: an epidemiological analysis from 2008 to 2021”.^1^ They suggest that our results may lead to an underestimation of the number of deaths reported in our study. However, we disagree, as our study aimed to “analyze the number of hospitalizations, the length of hospital stay, and mortality due to asthma”. Therefore, it could not underestimate the total number of deaths in Brazil. In addition, we utilized the Information Technology Department of the Brazilian Unified Health Care System (DATASUS), a secure public platform provided by the Ministry of Health, which specifically reports the mortality of hospitalized patients, as clearly stated in our study. The results presented by Antunes et al. are not contradictory but rather complementary. For instance, while their findings highlight an increase in the number of deaths, this observation alone raises concern without explaining the underlying causes. On the other hand, our findings on the reduced mortality in hospitalized patients strongly suggests that the increased mortality may be due to inadequate pharmacological treatment for those not hospitalized.

We acknowledge that there is a discrepancy in the number of deaths reported in 2013 in our study compared to the findings of Cardoso et al.^2^ However, we emphasize that this comparison is inappropriate, as they are analyzing entirely different populations. Furthermore, our study indicated in the limitations section that the data analyzed were collected from electronic records, which may vary from other sources. Regarding the pandemic period, we also noted that the reduction in the number of hospitalizations from 2020 to 2021 could be associated with the restriction measures recommended by the World Health Organization (WHO) due to COVID-19.

In addition, the *Sociedade Brasileira de Pneumologia e Tisiologia* recently published data regarding asthma in Brazil (also collected from DATASUS), indicating that in 2022, there were 83,155 hospitalizations due to asthma and 524 deaths. The report states that the “extension of care and access to medications… led to a significant drop in asthma medication”.^3^ Consistent with our findings, the authors also assert that the “expansion of care and access to medications led to a significant drop in hospitalizations for asthma”.^1^ We agree with the authors that it is crucial for future epidemiological studies on asthma mortality to employ standardized methods and reliable data sources. Finally, contrary to the statement made by Antunes et al., the number of patients was standardized according to population growth based on data from the *Instituto Brasileiro de Geografia e Estatística* (IBGE, Brazilian Institute of Geography and Statistics) website. Therefore, we look forward to carefully analyzing the results that will be published by Antunes et al., as they will contribute to a better understanding of asthma mortality in Brazil.

## References

[1] Pinheiro DHA, Souza JVH, Justo AFO, Carvalho-Pinto RM, Lima FF, Carvalho CRF (2024). Asthma in the Brazilian Unified Health Care System an epidemiological analysis from 2008 to 2021. J Bras Pneumol.

[2] Cardoso TA, Roncada C, Silva ERD, Pinto LA, Jones MH, Stein RT (2017). The impact of asthma in Brazil a longitudinal analysis of data from a Brazilian national database system. J Bras Pneumol.

[3] Sociedade Brasileira de Pneumologia e Tisiologia (SBPT) (c2024).
